# Does Antigen Masking by Ubiquitin Chains Protect from the Development of Autoimmune Diseases?

**DOI:** 10.3389/fimmu.2014.00262

**Published:** 2014-06-03

**Authors:** Robert Weil

**Affiliations:** ^1^Unité de Signalisation Moléculaire et Activation Cellulaire, CNRS URA 2582, Institut Pasteur, Paris, France

**Keywords:** autoimmunity, ubiquitin, UBLs, immune tolerance, apoptosis, autophagy

## Abstract

Autoimmune diseases are characterized by the production of antibodies against self-antigens and generally arise from a failure of central or peripheral tolerance. However, these diseases may develop when newly appearing antigens are not recognized as self by the immune system. The mechanism by which some antigens are “invisible” to the immune system is not completely understood. Apoptotic and complement system defects or autophagy imbalance can generate this antigenic autoreactivity. Under particular circumstances, cellular debris containing autoreactive antigens can be recognized by innate immune receptors or other sensors and can eventually lead to autoimmunity. Ubiquitination may be one of the mechanisms protecting autoreactive antigens from the immune system that, if disrupted, can lead to autoimmunity. Ubiquitination is an essential post-translational modification used by cells to target proteins for degradation or to regulate other intracellular processes. The level of ubiquitination is regulated during T cell tolerance and apoptosis and E3 ligases have emerged as a crucial signaling pathway for the regulation of T cell tolerance toward self-antigens. I propose here that an unrecognized role of ubiquitin and ubiquitin-like proteins could be to render intracellular or foreign antigens (present in cellular debris resulting from apoptosis, complement system, or autophagy defects) invisible to the immune system in order to prevent the development of autoimmunity.

The central tolerance occurs in the thymus, where negative selection eliminates most of the developing thymocytes that can recognize self-antigens. Peripheral T cells recognize antigens that have been processed and presented in association with the major histocompatibility complex (MHC) by the antigen presenting cells. In addition to central tolerance, several peripheral tolerance “checkpoints” act to prevent self-reactivity. Indeed, self-reactive T cells can be suppressed by regulatory T cells, eliminated by clonal deletion or inactivated by a state of unresponsiveness known as T cell anergy. In T cells, activation-induced cell death (AICD) is responsible for maintaining tolerance to self-antigen. Pathogen invasion gives rise to massive immune cell proliferation until the infection is resolved and excess of immune cells is also eliminated by activation-induced cell death (AICD) via an apoptotic Fas (CD95; APO-1) or a tumor necrosis factor-related apoptosis-inducing ligand (TRAIL)-dependent pathway. Failure of these processes (inactivation mutations or decrease expression of CD95, Fasl, or other components of the Fas signaling pathway) can lead to lymphoproliferation and autoimmunity (1–4).

The immune system can be classified in innate and adaptive immunity. Innate immunity is a non-specific defense mechanism that starts immediately or within an hour in response to pathogens. The cells of the innate system play an important role for the initiation of the adaptive immune responses, which is also known as the acquired immune system. This system is more sophisticated and specific and is composed of the humoral (production of antibodies by B lymphocytes) and the cellular immunity (clonal expansion of specific T lymphocytes such as cytotoxic T lymphocytes).

The innate functions can be divided into six steps: migration, recognition, phagocytosis, antigen processing, presentation of the antigenic peptide to lymphocytes, and cytokine secretion. There are different possibilities for a dying cell or a pathogen to be eliminated from the body. Cells that undergo programed cell death called apoptosis can set up an initiation signal (“eat me signal”) to allow their recognition and digestion by phagocytic cells (5–11). Several reports have shown that the deposition of proteins of the complement system on apoptotic cells following the activation of the complement pathways is required for their efficient digestion by macrophages (12–15).

Selective autophagy of pathogens, organelles, and protein complexes represents an important host innate mechanism that allows their removal from the body. As for apoptosis, these processes require the modification of these pathogens, organelles, and protein aggregates by an “eat me signal,” which involves a complex modification of the bacteria, organelles, or protein complexes to allow their recognition by specific cargo receptors (16–25).

We will see that improper removal of dying cells or pathogens can cause autoimmune diseases.

## Role of Defective Apoptosis, Cellular Uptake by Complement, or Autophagy in Autoimmunity

### Apoptotic- and complement-mediated cellular uptake defects and autoimmunity

In adult tissues, cell death compensates for cell division. Apoptosis (Greek for “falling off”) also known under the name of programed cell death is a form of cell death, which presents specific morphological changes. Apoptosis can be mediated by the activation of cell death receptors on their cell surface such as Fas or by intracellular inducers such as Staurosporine (26–28). The intramolecular mechanism responsible of apoptosis involves a family of proteases called Caspases that have a Cysteine at their active site and cleave their substrates at specific Aspartic acids (29–33). Caspases are synthesized as inactive precursors (pro-Caspases), which are usually activated by cleavage by other Caspases. Caspases 8, 9, and 3 play pivotal functions in apoptotic cells. Caspases 8 and 9 activate Caspase 3 that subsequently cleaves vital substrates such as nuclear lamins or DNase leading to the breakdown of nuclear lamina and fragmentation of DNA. Mitochondria play an important role in apoptosis through release of cytochrome *c*. Cell death can be regulated by a complex network of pro- and anti-apoptotic proteins such as p53, which act to regulate the expression of death receptors and the mitochondria outer membrane Bcl2 family of proteins, which are involved in the Caspase activation pathways. Bcl2 and Bcl-XL inhibit apoptosis by preventing the release of cytochrome *c* from the mitochondria (34), whereas others like Bax and Bak stimulate the release of cytochrome *c* from mitochondria (35). Bax and Bak are themselves activated by other proteins belonging to the Bcl2 family such as Bid (35). A third group of factors, the inhibitors of apoptosis (IAP), consists of structurally conserved proteins (XIAP, cIAP1, c-IAP2, XIAP, livin α and β, ILP2, and survivin) that can block apoptosis through their inhibitory interaction with specific Caspases (36, 37). Several IAP proteins have been shown to regulate apoptosis in a Caspase-independent manner through the JNK or NF-κB signaling pathways (38–40). Interestingly, IAPs function as E3 ubiquitin ligases and can target cellular proteins for proteasomal degradation, this process being essential for apoptosis (41). IAPs activities are regulated by second mitochondria derived activator of Caspases (smac) (42). Signals from dying cells such as expression of phosphatidylserine at cell surface can be recognized by multiple receptors of macrophages or dendritic cells and defects in the clearance of apoptotic cell debris or in the uptake of dying cells can lead to autoimmunity (43, 44).

The complement system is composed of 30 different proteins that are either circulating in the serum or attached to the cell surface. This system plays four major functions: lysis of pathogens, activation of inflammation, opsonization, and immune clearance. For example, cellular uptake can be mediated by macrophage-associated complement receptors that constitute susceptibility genes for the development of the autoimmune disease systemic lupus erythematosus (SLE) (13, 45–49). Interestingly, it has been proposed that inadequate clearance of apoptotic cells due to the reduced level of complement is responsible for these diseases (50). Once engulfed, antigens derived from dead cells are processed and presented at the cell membrane in association with the MHC. These MHC/Ag interactions subsequently stimulate T helper cells that can release cytokines such as interferon α/β (IFN) to activate macrophages, monocytes, and B cells. Defects in the clearance of apoptotic cell or in the uptake of the dying cell have been linked to autoimmune diseases (51–54). Cell debris can be recognized by innate immune receptors or other sensors to develop autoimmunity.

### Autophagy defects and autoimmunity

Autophagy (Greek for “self-eating”) is an evolutionary conserved mechanism that was first described by Christan de Duve as a lysosome-mediated degradation process for damaged cytoplasmic constituents. In macroautophagy, a double membrane called “phagophore” forms the autophagosome that surrounds cytoplasmic proteins or organelles. Autophagosome then fuses with lysosomes to create autolysosomes in which the cytosolic cargos are degraded. Autophagosome formation requires evolutionarily conserved proteins known as Atg proteins in yeast. Microtubule-associated protein light chain 3 (LC3) is the mammalian homolog of yeast Atg8 and is a widely used marker of autophagy. LC3 is localized in autophagosomes and the amount of its phagosome-associated form, named LC3-II, is correlated with the amount of autophagosome formed. Autophagy also contributes to innate immunity by protecting host cells from invading pathogens, a process called xenophagy. Substrates for selective autophagy are recognized either directly or indirectly (through “eat me” signals) in the cell (see below). As for apoptosis, autophagy imbalance – i.e., perturbation of autophagy function or autophagy gene defects – has been involved in autoimmune diseases (55–58).

## Ubiquitin as a “Camouflage Uniform” to Avoid Recognition of Antigens by the Immune System?

**Figure 1:**
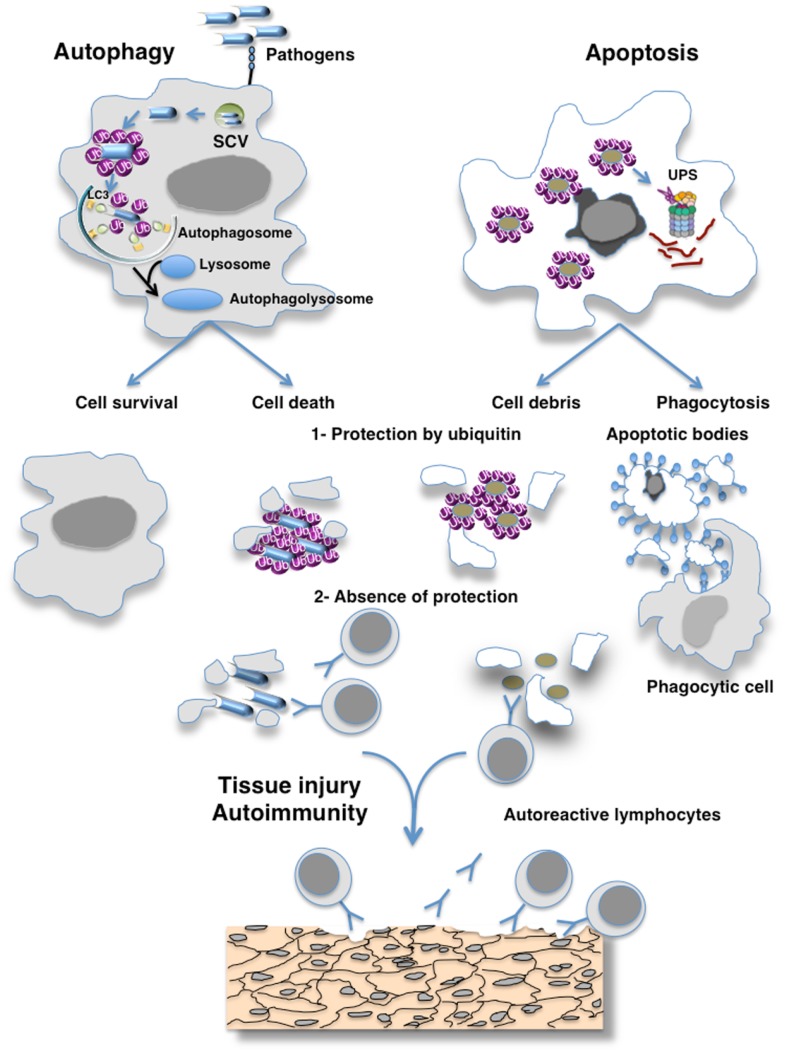
**Hypothesis of the masking of epitopes in cell debris by ubiquitin preventing their recognition by the immune system**. Eukaryotic cells use autophagy and the ubiquitin–proteasome system (UPS) as their major protein degradation pathways. Whereas the UPS is involved in the rapid degradation of proteins, autophagy pathways can selectively remove protein aggregates, damaged or excess organelles, and pathogens. Ubiquitin have been involved as a specific factor for selective autophagy as exemplified here by autophagy of pathogens. Different cellular adaptors connect pathogens to the protein light chain 3 (LC3), a key autophagy-related protein that is located at the surface of autophagosomes. Proteasome-mediated degradation also requires the ubiquitination of the cargo, which is then recognized by ubiquitin receptors allowing their degradation by the 26S proteasomes. The defective clearance of apoptotic debris by phagocytes and autophagy imbalance can result in the accumulation of cell debris that is responsible for the initiation of systemic autoimmunity. Such defect of clearance induces the release of immunogenic intracellular contents from the dying cells. I hypothesize that ubiquitination protects antigens generated by cells escaping from destruction by the immune system and that failure of ubiquitination mechanisms may induce an immune response to cross-reactive self-antigens that can lead to organ damage.

My reflection was guided by different publications on the biological properties of ubiquitin chains (abundance, structure, immunogenicity, and function), E3 ubiquitin ligases and deubiquitinases (Figure 1).

Ubiquitin, a peptide of 76 amino acids, can be covalently attached to protein substrates on lysine residues either as a monomer or polymer. Its amino acid sequence is highly conserved with little variance from insects to human. These ubiquitin chains are covalently attached to protein substrates by the concerted action of enzymes called E1, E2, and E3. Ubiquitin chains can be formed by isopeptide linkages between one of the seven internal lysine residues of an ubiquitin moiety and the carboxy-terminal residue of another ubiquitin. More recently, it has been shown that ubiquitin chains can also be formed in a head-to-tail fashion by peptidic bonds between the C-terminal glycine and the amino-terminal methionine of consecutive ubiquitin molecules. These ubiquitin modifications are reversible since deubiquitinating (DUB) enzymes can remove them.

### Ubiquitin chains possess all of the requirements for masking antigens to the immune system

#### High abundance

Ubiquitin is highly abundant in all eukaryotic cells and tissues. It is the second most common post-translational protein modification after phosphorylation. Mass spectrometry analyses identified around 20,000 ubiquitination sites present on more than 5000 ubiquitin putative substrates (59–61).

#### Low immunogenicity

Importantly, ubiquitin is a highly conserved polypeptide and is poorly immunogenic (62). Interestingly, the presence of antibodies to ubiquitin has been found in 80% of patients exhibiting autoimmune responses associated with SLE and in those presenting a systemic sclerodermia (they are only present in 3% of normal human sera) (63–65). These reports suggest that the accumulation of ubiquitinated proteins is highly immunogenic in these autoimmune patients. However, this could not been confirmed by another study (66).

#### Conformation

The important variations in length and linkage architecture of these ubiquitin chains may provide a canvas surrounding the substrate that can adopt an open or more compact conformation depending on the type of ubiquitin chain and can determine the fate of conjugated proteins (67). Furthermore, polyubiquitin chains containing different linkages within the same chain (mixed chains) have also been described.

#### Functions

Since the discovery that ubiquitination targets proteins to proteolysis by Aaron Ciechanover, Avram Hershko, and Irwin Rose, it has been shown that ubiquitin conjugation controls different cellular processes (68).

The attachment of one or more ubiquitin provides a large interaction surface and results in a vast number of potential signals depending on the various conformations adopted by the ubiquitin chains. In addition to their role in targeting proteins for proteasomal and lysosomal degradation, signaling roles of ubiquitin have been discovered in many processes such as endocytosis, DNA repair, autophagy, and NF-κB activation (69–72).

Autophagy and the ubiquitin–proteasome system (UPS) are the two major intracellular degradation pathways. While the UPS is widely known for its role in intracellular protein degradation, autophagy is responsible for the degradation of long-lived proteins, organelles, and bacteria (71). Increased transcription of the *ubiquitin* gene that leads to ubiquitination of cellular proteins and degradation by the UPS is also characteristic of apoptotic cell and is required for the apoptotic process (73). The ubiquitin system is also widely used for autophagy. Various cytoplasmic bacteria are targeted for xenophagy through ubiquitin-mediated pathway. Bacteria and autophagosomes decorated with polyubiquitin chains recruit ubiquitin-binding adaptors, which in turn engage the autophagic machinery to restrict the proliferation of the bacteria (74). Two E3 ligases responsible for linking ubiquitin to the bacteria and/or bacteria-containing phagosomes have been recently identified as LRSAM1 and Parkin (75, 76). Ubiquitination of these aggregates and pathogens triggers the recruitment of different selective receptors, which in turn target these materials to the autophagosome membrane through their binding to autophagy modifiers such as LC3-like molecules that are present at the surface of autophagosomes (71). Damaged mitochondria are directly targeted to LC3-like molecules by the selective receptors Nix and FUNDC1 (16, 17). Four other autophagy receptors recognize ubiquitin chains that behave as “eat me signal” through their ubiquitin-binding domain (UBD): p62 (SQSTM1), NBR1, NDP52, and optineurin (16–25). These receptors also bind to LC3-like molecules through short LC3-interacting regions (LIRs). The sequential recruitment of DUB enzymes may negatively regulate the autophagy process. The physiological relevance of the cargo receptors is underscored by the presence of mutations in p62 and optineurin genes found in human patients with Paget’s disease of bone, primary open angle glaucoma, amyotrophic lateral sclerosis, or hepatocellular carcinoma (77–82).

In addition to phagocytosis and autophagy, neutrophil extracellular trap (NET) consisting of microbicidal molecules and extrusion of decondensed chromatin has been shown to be important strategy by which neutrophils kill microorganisms (83, 84). This process called NETosis is enhanced in SLE (85–87). A proposed hypothesis is that NETs may provide neoantigens for autoantibody formation (66, 88–91). Post-translational modifications of NET proteins such as ubiquitination and their proteolytic cleavage by NET proteases may provide the formation of neoantigens in certain circumstances. This could provide an explanation for the immunogenicity of ubiquitin in SLE and sclerodermia and in the rupture of tolerance responsible for these pathologies (see above).

It is well known that pathogens are involved in the appearance of autoimmune diseases (92), however, it is unclear whether the detection of microbial antigens by immune cells could result from a defects of masking of the antigens. Nethertheless, the detection of microbial antigens drive the clonal proliferation of specific T and B cells, and multiple mechanisms have been proposed to explain how pathogens can induce the expansion of autoreactive cells (93, 94). Among these mechanisms, it has been suggested that pathogenic antigens might react with the self and provoke tissue damage, a process known as molecular mimicry (94). Other hypotheses have been proposed to explain the emergence of autoreactive cells, including bystander activation of autoimmune cells caused by an inflammatory environment, processing and presentation of cryptic antigens, or adjuvant effects of pathogens.

I propose that, in addition to their roles in targeting proteins for degradation or cell signaling, and pathogens for destruction ubiquitin may protect antigens in cell debris resulting from apoptotic and autophagy defects and in NETs from recognition by the immune system (Figure 1).

In this hypothesis, we should expect that alteration of the ubiquitin machinery would lead to autoimmunity. Consistently, several mechanisms clearly illustrate the link between the ubiquitin system and peripheral tolerance mechanisms. These mechanisms include homeostatic regulation, T cell apoptosis, anergy, and regulatory CD4^+^ T cells. As described above, apoptosis is a highly regulated process involving the transcription of ubiquitin. In addition to being involved in T cell apoptosis, ubiquitin is also implicated in the molecular mechanism of T cell anergy. Clonal T cell anergy is a tolerance mechanism in which T cells are unresponsive to a second stimulation of the TCR. T cells can become anergic after encountering antigen in the absence of a CD28 co-stimulation or interleukin-2 (IL-2). The characteristic feature of clonal T cell anergy is a decrease in cytokine production and proliferation. The signaling cascade that leads to clonal T cell anergy has been the focus of many investigations and it has been shown that E3 ubiquitin ligases modulate different pathways leading to anergy. The transcription factor nuclear factor of activated T cells (NFAT) is a crucial factor for the induction of T cell anergy. In T cells, CD28 co-stimulation activates AP1-family of transcription factors, which cooperates with NFAT to induce the expression of genes encoding effectors of the T cell activation pathway. In contrast to this situation, a transcriptional program of anergy can be activated by NFAT in the absence of AP1 (95).

Interestingly, E3 ligases are over expressed in anergic T cells (96, 97) and among other anergy-associated genes, NFAT induces the transcription of E3 ligases genes (95). NFAT binds to promoter/enhancer sequences of gene related to anergy in lymphocytes (GRAIL), Cbl-b, Itch. Interestingly, the protein growth response (Egr)-2 and Egr-3 of the early Egr family of transcription factor are induced in response to NFAT and control the expression of Cbl-b and inhibition of T cell activation (98, 99). Consistently, it has been shown that Erg3^−/−^ mice have increased susceptibility to autoimmunity (99). The upregulation of the anergy related genes were abrogated by cyclosporine A, an inhibitor of the calcium-dependent serine-threonine phosphatase calcineurin, which promotes the activation of NFAT. Since the discovery of the increased expression of E3 ligases in anergic T cells, the role of E3 ligases in peripheral T cell tolerance has been the focus of many reviews (100–104). Knocked-out mice and those carrying mutations or overexpressing ubiquitin ligases and deubiquitinases [such as autoimmune regulator (AIRE), Itch, Nedd4, Roquin, Cbl-b, TNFR-associated factor 6 (TRAF6), Act1, Peli1, NEDD4-family interacting protein 1 (Ndfip1), A20, CYLD] develop autoimmune diseases (100–102, 105–107). In particular, upregulation of the E3 ligases Cbl-b, GRAIL, Itch, and Peli1 during immune tolerance have been involved in the ubiquitination of key signaling molecules of the TCR pathway, and these studies have highlighted the key regulatory role of ubiquitin in the induction of tolerance and prevention of autoimmunity (100–102).

## E3 Ubiquitin Ligases and Immune Tolerance

### Cbl-b

The RING-type E3 ligase Cbl-b (Casitas B cell lymphoma b) was the first E3 ligase implicated in T cell tolerance (97, 105, 108). It belongs to a family of proteins comprising three members: c-Cbl, Cbl-b, and Cbl-3. Cbl-b is a critical regulator of T cells since Cbl-b-deficient T cells do not require CD28 co-stimulation for IL-2 production and proliferation. This observation suggests that Cbl-b regulates CD28-dependent T cell activation. Additionally, the loss of Cbl-b also results in aberrant activation of NF-κB in response to TCR stimulation. This hyperactivation of NF-κB is mediated by PKCθ, which promotes the formation of a complex formed by three signaling proteins, Carma1, Bcl10, and Malt1 as known as the CBM complex (109). Interestingly, the dysregulation of Cbl-b pathway in mice is responsible for increased susceptibility to experimental autoimmune diseases (96, 97, 108). The mechanism used by Cbl-b to downregulate T cell activation involves the GTP exchange factor Vav and the p85 subunit of phosphatidylinositol 3-kinase (PI3K). Previous studies have shown that PI3K phosphorylates phosphatidylinositol-4,5-diphosphate (PIP2) at the D3 position to form active lipid second messenger that regulate the exchange activity of Vav. It has been reported that Cbl-b induces the attachment of K48-linked ubiquitin chains to p85 and its proteasomal degradation and thus indirectly regulates Vav activation. The absence of Cbl-b increases CD28-mediated Vav1 activation and cytoskeleton reorganization (96, 97, 110).

Cbl-b constitutively interacts with the p85 subunit of the lipid kinase PI3K through its Proline rich region (111). Then, Cbl-b promotes p85 ubiquitination and affects its recruitment to the immune synapse, preventing the interaction of PI3K with CD28 (112). Finally, it has been shown that Cbl-b contributes to the disintegration of the immune synapse upon anergy induction (113).

### TNFR-associated factor 6

TNFR-associated factor 6 is an adaptor protein that can act as an E3 ubiquitin ligase to mediate the activation of the NF-κB signaling pathway in response to TNF or interleukin-1 (IL-1)/TLR family members. Interestingly, TRAF6 expression is upregulated in activated T cells (114).

Unlike Cbl-b, TRAF6-mediated ubiquitination is associated with protein activation independently of the proteasomal degradation pathway. TRAF6 mediates the attachment of K63-linked ubiquitin chains to important signaling proteins allowing the activation of these pathways. In addition, TRAF6-deficient mice created by complementation of Rag2^−/−^ blastocysts developed a progressive inflammation disease characterized by hyperactivation of CD4^+^ T cells. Moreover, T cell-specific deletion of TRAF6 (*Traf6*-ΔT) resulted in multiorgan inflammatory disease and resistance of T cells to the suppressor function of CD4^+^CD25^+^ regulatory T cells (114). Like Cbl-b^−/−^ T cells, naïve *Traf6*-ΔT T cells hyperproliferate in response to anti-CD3 stimulation. Importantly, TRAF6-deficient cells exhibit hyperactivation of the PI3K-Akt pathway, suggesting a negative regulatory role of TRAF6 in CD28-dependent T cell activation. Consistently, as for Cbl-b, loss of TRAF6 restores the ability of CD28^−/−^ T cells to proliferate and produce IL-2, suggesting that TRAF6 is a critical mediator of peripheral tolerance (115). TRAF6 also plays a role in thymic development since its deficiency results in disorganized distribution of medullary thymic epithelial cells (mTECs) and in the absence of mature mTECs (116). The grafting of TRAF6^−/−^ thymic stroma tissue into athymic nude mice induced autoimmunity.

### Gene related to anergy in lymphocytes

Gene related to anergy in lymphocytes (GRAIL) is a RING E3 transmembrane glycoprotein that localizes to vesicular structures in the cell. GRAIL contains a single transmembrane-spanning domain that promotes its endosomal subcellular localization, a RING domain, a protease-associated (PA) domain, and a coiled-coil region (117). GRAIL was discovered using differential display to examine early changes in gene expression in anergic conditions (117). Retrovirally transduced T cell hybridoma that expresses GRAIL strongly decreases IL-2 and IL-4 production and inhibits proliferation in response to anti-CD3 and anti-CD28 co-stimulation. This function of GRAIL is dependent on endosomal trafficking, which suggests that GRAIL may target a protein of the endocytic pathway to control cytokine production. It was shown later using yeast two-hybrid system that GRAIL functionally interacts with two isoforms of the ubiquitin-specific protease Otubain 1 that belongs to the ovarian tumor (OTU) superfamily (118). Otubain 1 expressing cells contain less amount of GRAIL and secrete large amount of IL-2 following antigenic stimulation, while those expressing the alternatively spliced isoform, Otubain 1 alternative reading frame 1 (ARF-1), contain an increased amount of GRAIL and are functionally anergic. These data further demonstrate that the two isoforms of Otubain 1 have opposing effects on GRAIL and that Otubain 1 ARF-1 recruits the ubiquitin-specific protease 8 (USP-8) to promote GRAIL deubiquitination and stabilization. Using a prokaryotic system developed to screen for E3 ligase substrates, Rho guanine dissociation inhibitor (RhoGDI) was found as a potential substrate of GRAIL (119). GRAIL attaches ubiquitin-linked chains to RhoGDI and inhibits its effect on the reorganization of the cytoskeleton in T cells (120). Importantly, GRAIL-deficient mice are resistant to immune tolerance induction and exhibit a greater susceptibility to autoimmune diseases. GRAIL promotes CD3 ubiquitination and consequently, GRAIL-deficient T cells fail to regulate TCR expression in response to TCR stimulation and have an enhanced activation of NFATc1, while T cells expressing GRAIL present an enhanced TCR downregulation (107). It has been suggested that GRAIL is also responsible for the decrease cell surface expression of CD40L that occurs following anergy induction of CD4^+^ T cells (121). Furthermore, similarly to CD40L^−/−^ mice, GRAIL overexpression results in reduced lymphoid follicle formation. GRAIL also targets CD83, a costimulatory signal for T cell proliferation and function, for its degradation by the proteasome 26S (122).

### Itch

The E3 ligase Itch is encoded by the *agouti* locus, and its mutation is responsible for constant itching of the skin and development of a systemic lymphoproliferative disease characterized by enlarged secondary lymphoid organs (123). In addition, Itch^−/−^ T cells present an activated phenotype and enhanced proliferation (124). CD4^+^ T cells of these mice are also resistant to Treg-dependent immunosuppression (125). Unlike Cbl-b and GRAIL, Itch contains a HECT domain that is responsible for its E3 ligase activity. Itch also includes a C-terminal C2 domain, which promotes its localization to endosomes. Itch expression is upregulated under anergizing stimuli allowing Itch to target members of the Jun family of transcription factors (i.e., c-Jun, Jun B) for ubiquitination and subsequent proteasomal degradation (126, 127). Since JunB is required for T helper 2 (Th2) differentiation, Itch downregulation in lymphocytes causes aberrant Th2 differentiation (124). Furthermore, Itch is responsible for the monoubiquitination of PLC-γ1 and PKC-θ and their lysosomal degradation (113). In these conditions, decreased PLC-γ1 expression is responsible for impaired Ca^2+^ signal and decreased stability of the immune synapse.

### NEDD4-family interacting protein 1

NEDD4-family interacting protein 1 (also known as N4WBP5) is upregulated in activated T cells. Ndfip1 interacts with NEDD4-family members and is proposed to function as an adaptor for ubiquitinated targets of the NEDD4-family such as Itch (128–130). Ndfip1 associates with Itch and promotes JunB degradation. Importantly, Ndfip1 deficiency is responsible for the failure of peripheral CD4^+^ T cell tolerance to self and innocuous foreign antigen, forcing them to exit cell cycle after a few divisions. This mechanism prevents CD4^+^ cells from differentiating into IL-4 producing cells. Ndfip1 deficiency disrupts peripheral T cell tolerance to pancreatic islets and increases the incidence of autoimmune pancreatic destruction and diabetes (131).

### Roquin

Roquin was identified as a novel RING finger E3 ubiquitin ligase in a systematic screen using ethylnitrosourea (ENU)-induced mutation in the mouse and screening for autoimmunity (132). The first mutation identified was named *sanroque* because the accompanying lymphadenopathy exhibited all the features of SLE and small intestine inflammation: antibodies against dsDNA, proliferative glomerulonephritis with deposition of immune complexes necrotizing hepatitis, anemia, and autoimmune thrombocytomia (132, 133). Using a genetic approach, it was found that the *sanroque* mutation corresponded to a missense mutation of the *Roquin* gene. At the cellular level, these mice present increased numbers of germinal centers and follicular helper T cells. *Sanroque* CD4^+^ T cells express high level of the CD28 paralog “T cell costimulatory receptor inducible T cell costimulator” (ICOS) that was reduced upon re-expression of Roquin. Roquin was shown to localize in cytoplasmic stress granules (P bodies) (134) and to limit ICOS expression by promoting the degradation of ICOS mRNA. A conserved segment containing a region complementary to T cell expressed microRNA in ICOS 3′ untranslated mRNA was shown to be critical for the regulation by Roquin (135). However, instead of using microRNAs, a trimolecular complex containing Roquin, the RNA helicase Rck and the enhancer of decapping Edc4 was shown to promote ICOS mRNA decapping and ICOS repression (136).

To test the participation of ICOS with the *sanroque* phenotype, *sanroque* mice were crossed with ICOS^−/−^ mice (135). Interestingly, the partial reduction of ICOS expression was accompanied by a reduction of lymphadenopathy, splenomegaly, total T- and B-cell number, and germinal center B cell number thus demonstrating that overexpression of ICOS contributes to *sanroque* mice autoimmune phenotype. Interestingly, it was further shown that tissue-specific ablation of Roquin in T, B cells, or in the entire hematopoietic system does not cause autoimmunity, while enforced Roquin expression in T cells exacerbates the severity of experimental arthritis (137). Finally, it was clearly shown that Roquin-1 has redundant function with Roquin-2 in the post-transcriptional repression of ICOS mRNA and that Roquin-2 compensates for the absence of Roquin-1, but not for its mutation (138, 139). It has been shown recently that Roquin-2 promotes ubiquitin-mediated proteasomal degradation of apoptosis signal-regulating kinase 1 (ASK1), a protein involved in the activation of JNK and p38 in response to stress (140). However, further investigation is required to define the ubiquitinated substrates of Roquin-1 and Roquin-2 that could explain the role of these proteins in autoimmunity.

### Autoimmune regulator

Autoimmune regulator (AIRE) gene mutation is responsible for the development of autoimmune-polyendocrinopathy-candidiasis ectodermal dystrophy (APECED), also known as autoimmune polyglandular syndrome type 1 (APS1), an organ-specific autoimmune disease. Mice carrying a defective AIRE gene also develop autoimmunity (141, 142). The clinical course in human and mice appears after a latent period. This period can be reduced in mice by the cross breeding of AIRE-deficient mice with Cbl-b KO mice (143). AIRE is predominantly expressed in medullar epithelial cells of the thymus and is considered to play important roles in the establishment of self-tolerance. mTECs have been implicated in the clonal deletion or inactivation of self-reactive thymocytes. Many ectopically expressed antigens are associated with organ-specific autoimmune diseases and it has been shown that AIRE-deficient mTECs present a decrease in the ectopic transcription of genes encoding peripheral antigens (141). Interestingly, AIRE functionally interacts with the small ubiquitin-related modifier (SUMO) ligase PIAS1 in the nuclear bodies, which cooperates to activate AIRE-known target genes (144). AIRE also functions as an E3 ligase. The AIRE gene is composed of two PHDS and SAND domains and its E3 ligase activity is mediated by the PHD1 domain and abolished by disease-causing mutations in the PHD1 (C311Y and P326Q) (145).

### NF-κB activator 1

NF-κB activator 1 (Act1) was discovered by searching for potential genes that play a role in NF-κB activation and was cloned in parallel via a yeast two-hybrid screen using NEMO as bait (146, 147). The structure of Act1 consists of two TRAF binding domains, an U-Box E3 ligase, a helix-loop-helix (HLH) and a SEF/IL-17R (SEFIR) domain. Act1 is an important negative regulator of B cell-mediated humoral immune response through its function in CD40 and BAFF signaling (two TNF receptor superfamily members). Upon CD40 stimulation, Act1 is recruited to CD40 that also interacts with TRAF3 (148). CD40 and BAFFR play critical roles in B cell survival and maturation, and dysregulation of these pathways leads to autoimmunity. In agreement with Act1 B cell function, Act1 knocked-out mice developed B cell-mediated autoimmune phenotypes including increased peripheral B cells, lymphadenopathy and splenomegaly, hypergammaglobulinemia, and autoantibodies (149). Interestingly, it was recently shown that crossing of AM14 transgenic (Tg) rheumatoid factor mice to Act1^−/−^ mice leads to the activation of AM14 Tg B cells. AM14 Tg Act1^−/−^ mice developed enlarged spleens and lymph nodes and presented expansions of rheumatoid factor-specific autoreactive B cells (150).

CD4^+^ T helper cells are divided into two lineages: T helper 1 (Th1) that secretes IFNγ and Th2 cells that secrete IL-4, IL-5, and IL-13. Recently, a third lineage that secretes IL-17 (Th17) has been found to play an important role in the defense against bacteria and fungal infections. The number of these cells is increased in autoimmune diseases. Act1 is a critical mediator of IL-17 signaling and has been involved in this pathway because its SEFIR domain is closely related to IL-17-receptor SEFIR domain. In response to IL-17, Act1 interacts with IL-17 receptor through an homotypic SEFIR–SEFIR interaction. Following this recruitment, Act1 attaches K63-linked ubiquitin chains to TRAF6 allowing its interaction with the TGFβ activated kinase 1 (TAK1) and subsequent phosphorylation of the NEMO/IKK complex followed by NF-κB activation. Because IL-17 is important in experimental autoimmune encephalomyelitis (EAE) pathogenesis, a model of multiple sclerosis, the effect of Act1 was assessed on a mouse model of EAE. Interestingly, Act1-deficient mice showed a delay in the onset of neurological impairment and had much lower severity compared to wild-type mice (151).

### Pellino 1

The mammalian Peli (Pellino) family is composed of three members, Peli1, Peli2, and Peli3. The E3 ubiquitin ligase activity of Peli proteins is dependent on their C-terminal RING domain. Peli1 is essential for the TLR-mediated NF-κB activation dependent on the adaptor TRIF (152). Peli1-deficient T cells are hyper responsive to TCR and CD28 signals, they secrete more IL-2 and their naïve CD4^+^ T cells proliferation is not inhibited by Treg cells as opposed to wild-type CD4^+^ T cells (105). Consequently, Peli1^−/−^ mice develop autoimmunity such as enlarged peripheral lymph nodes, moderate splenomegaly, and infiltration of many organs by cells from the immune response. Consistently, T cells from Peli1^−/−^ mice show more pathogenic potential in EAE. In addition, Peli1 deficiency causes hyperactivation of late phase NF-κB and impairs ubiquitination and degradation of c-Rel, which is important for the thymic development of Tregs by directly inducing transcription of the Treg-specific transcription factor Foxp3 (153).

## Deubiquitinase and Immune Tolerance

The ubiquitin-editing enzyme A20 and the deubiquitinase CYLD are important negative regulators of NF-κB signaling, and this control is important for adaptive and innate immunity. The modification of key signaling proteins such as NEMO, TRAF6, RIP1, Bcl10, MALT1 with K63-linked ubiquitin chains, or linear ubiquitin chains has emerged as an essential process for NF-κB activation. Ubiquitination can be reversed by DUB enzymes such as A20 and CYLD.

### A20

A20 encoded by the *TNF-α-inducible gene 3* (*TNFAIP3*) was identified in endothelial cells as a primary response gene induced upon treatment with TNF (154, 155). A20 was shown to be an ubiquitin-editing enzyme containing an amino-terminal DUB activity mediated by its OTU domain and a carboxy-terminal zing finger (ZnF) domain responsible for its E3 ubiquitin ligase activity (156). Further studies demonstrated a role of A20 not only in TNF signaling, but also in IL-1-, CD40-, TLR-, TCR-, and BCR-mediated NF-κB activation. A20 expression is rapidly induced upon NF-κB activation, which suggests that A20 prevents the persistent activation of NF-κB that could have negative effects on cell viability. In addition to ending NF-κB signaling, A20 exhibits NF-κB-unrelated functions. In response to pathogen invasion, A20 inhibits RIG-I-induced IRF activation and IFN responses by removing K63-linked polyubiquitin chains from the innate immune kinases TBK1 and IKKε (157–160). A20 has also been shown to control autophagy in response to TLR activation through the deubiquitination of Beclin 1, a protein essential for autophagy (161). In addition, A20 functions as an anti-apoptotic protein in several cell types and has been shown to cleave TRAIL-mediated ubiquitination of Caspase 8 in order to inhibit apoptosis (162).

Regarding autoimmunity, *A20* has been reported as a disease susceptibility gene for human inflammatory and autoimmune pathology, including rheumatoid arthritis (RA) and juvenile idiopathic arthritis, SLE, inflammatory bowel disease (IBD), celiac disease, psoriasis, type 1 diabetes, Sjogren’s syndrome, coronary artery disease, rheumatic heart disease, and systemic sclerosis (163). A case-control study in African-American SLE patients with a genetic polymorphism of the *A20* gene shows that it alters DUB activity and mediates risk of autoimmunity (164). As a consequence of the lack of regulation of NF-κB pathways, A20-deficient mice present severe inflammation and hypersensibility to TNF signaling and MyD88-dependent TLR signaling initiated by the commensal flora (165, 166). Cell type-specific deletion of A20 in B cells, dendritic cells, myeloid cells, intestinal epithelial cells, and keratinocytes confirmed that A20 plays a crucial role for the maintenance of tissue homeostasis and the control of systemic inflammation (167–173). Mice that specifically lack A20 in all cells of myeloid origin, develop spontaneous polyarthritis with the presence of type II collagen autoantibodies and inflammatory cytokines in serum (171). An *A20*-conditional KO in dendritic cells induces massive splenomegaly and lymphadenopathy. In one study, these mice developed an SLE-like phenotype, including the presence of double stranded DNA autoantibodies, glomerulonephritis, antiphospholipid syndrome, and arthritis, while, in an other study, they developed lymphocyte-dependent colitis, ankylosing arthritis, and enthesitis (172).

In B cells, A20 depletion induces enhanced B cell proliferation and survival as well as autoantibodies secretion (168, 169, 174). These mice exhibit a lupus-like autoimmune pathology characterized by increased numbers of germinal center B cells and glomerular immunoglobulin deposits. These mice also produce autoantibodies against cardiolipin, an important component of the inner mitochondrial membrane.

### Cylindromatosis

*Cylindromatosis (CYLD)* is a tumor suppressor gene whose mutations result in a predisposition to familial cylindromatosis, a disease characterized by the development of benign tumors of the skin. CYLD exhibits deubiquitinase activity and has been identified as a critical regulator of NF-κB signaling by different approaches (175–177). As for A20, CYLD synthesis is regulated by NF-κB. *CYLD* KO mice confirmed that CYLD is a negative regulator of NF-κB (178, 179). The role of CYLD in B cell function is controversial. Jin and colleagues found that *CYLD* KO mice present several abnormalities of the immune system such as enlarged lymph nodes, B cell hyperplasia, expansion of the B cell marginal zone and B cell hyper-responsiveness in response to BCR, or LPS stimulation (178). However, in other studies, CYLD deficiency did not affect peripheral B cell numbers, but increased NF-κB activation upon stimulation (180, 181). T cells derived from *CYLD* KO mice displayed an hyper responsive phenotype (179). Adoptive transfer of *CYLD* KO T cells into *RAG* KO mice that lack endogenous lymphocytes induced autoimmune symptoms and intestinal inflammation (179). To explore potential overlapping functions between A20 and CYLD, the *A20*/*CYLD* double KO in B cells was generated (181). Interestingly, the lack of CYLD did not exacerbate the developmental defects and hyper-responsiveness of the A20-deficient B cell activity. The expression of CYLD must be tightly regulated since overexpression of the short spliced variant of *CYLD* gene (*sCYLD*) resulted in splenomegaly and lymphadenopathy, hyperactivation of CD4^+^ T cells and decrease in mTECs. When these mice were crossed onto TCR Tg background, they developed colonic inflammation associated with high production of autoantibodies.

### Ubiquitin-like proteins and autoimmunity

Finally, ubiquitin-like proteins (UBLs) (182), such as SUMO and ISG15, which are proteins related to ubiquitin, might also protect their substrates from recognition by the immune system. Among SUMO paralogs, SUMO4 harboring the M55V polymorphism is associated with susceptibility to autoimmune diabetes (183), although it is not clear whether SUMO4 protein is expressed. Additionally, ISGylation affects many proteins that are localized in different cellular compartments, participates in various cellular processes, and also targets a number of viral proteins. Interestingly, a large-scale microarray study of muscle samples revealed that the autoimmune disease dermatomyositis was specifically associated with enhancement of ISGylation (184).

## Conclusion

Our understanding of the role played by ubiquitin in immune tolerance is still in its early stage. Although much attention focused on the function of E3 ligases and deubiquitinases in early events of T cell activation and immune tolerance, it is important to determine whether E3 ligase activity in dying cells could be involved in immune tolerance by preventing recognition by the immune system.

Another related issue is whether the UBDs that are present in a multitude of cellular proteins also protect proteins from recognition by the immune system through their interaction with ubiquitinated proteins. Intriguingly, defects in the interaction of ABIN1 with other proteins through its UBD cause autoimmunity in mice (185, 186). ABIN1 was originally identified by its interaction with the deubiquitinase A20, and mice with conditional knockout of *A20* developed autoimmunity (see above) (187). Human polymorphisms were also identified in the *ABIN1* coding gene, which constitutes a susceptibility gene for the development of autoimmune diseases.

In addition to changing the fate of proteins by targeting them to degradation via the proteasome pathway or to signaling complexes, ubiquitin may also mask epitopes that could lead to autoimmunity.

Finally, it is apparently difficult to conceive that the absence of ubiquitin ligase or deubiquitinases such as A20 or CYLD, which have the opposite effect leads to autoimmunity. However, it must be noted that the A20 and CYLD DUBs cleave K63-linked or linear ubiquitin chains, which are mostly important for protein–protein interactions. Consequently, their depletion increases the amount of K63-linked or linear-linked ubiquitinated proteins in signaling complexes and therefore enhances signaling. Consistently, the depletion of E3 ligases that mediate K48-linked ubiquitination of signaling proteins and proteasomal degradation also results in increased signaling. Furthermore, it is now well known that besides its DUB activity, A20 catalyzes the addition of K48-linked polyubiquitin chains to different substrates including Ubc13, UbcH5c, and RIP1 and targets them for proteasomal degradation (188) and that its depletion also impairs its E3 ubiquitin ligase activity. It is also important to consider that in lymphocytes, A20 is constitutively expressed to prevent uncontrolled activation of NF-κB and its proteasomal degradation or its cleavage by the paracaspase MALT1 impairs its inhibitory function to allow optimal NF-κB activation as exemplified by the increased antigen-mediated NF-κB signaling pathways in A20-deficient lymphocytes (189, 190). My hypothesis is that the decreased expression of E3 ubiquitin ligase induces autoimmunity by a dual mechanism: The loss of immune tolerance [absence of regulation of autoreactive lymphocytes or loss of regulatory T cell (Tregs) functions] and the absence of protection of epitopes against the immune system. However, we cannot make this assumption in the case of deubiquitinase deficiencies since epitopes are still protected by ubiquitin in these conditions.

In the past few years, it has been suggested that ubiquitin can be released from the cell to modulate the immune response (191). Ubiquitin is released from damaged erythrocytes, from damage tissues or from cells undergoing physiological turnover during prolonged blood storage. Besides its presence in the bloodstream, ubiquitin is also detectable in cerebrospinal fluid, bronchoalveolar lavage fluid, seminal plasma, and urine. Multiple diseases are known to be associated with increased concentration of extracellular ubiquitin. Its increased concentration in the serum has been reported in different pathologies including lupus erythematosus. Different properties have been attributed to extracellular ubiquitin such as antimicrobial activities. Intriguingly, ubiquitin have been originally purified from bovine thymus and characterized as a protein presenting a role in lymphocyte differentiation (192). Daily injection of ubiquitin induced T cell differentiation in the spleen and lymph nodes of athymic nu/nu mice. Interestingly, injection of ubiquitin into skeletal muscles led to a recruitment of lymphocytes (193). It has been suggested that ubiquitin presents immunosuppression activity against B and T cell functions (194). Despite the multiple effects that have been reported, little is known on the mechanism of action of extracellular ubiquitin. Recent data suggest that cellular uptake of extracellular ubiquitin is followed by its conjugaison to intracellular proteins (195, 196). Thus, the expression level of ubiquitin is not only controlled at the transcriptional level, but also by cellular uptake. Given that ubiquitin can be quickly available for protein modifications, that it lacks immunogenicity, that it attracts lymphocytes, and presents an immunosuppression activity, it is an attractive alternative hypothesis that post-translational modification of proteins with ubiquitin also neutralized their immunogenicity. Accordingly, administration of exogenous ubiquitin produces effects in various diseases including autoimmune diseases.

The experimental autoimmune EAE is a well-characterized model of the human autoimmune multiple sclerosis disease that is produced by the injection of brain extracts, which is responsible of demyelinisation. Using the same kind of approach, it would be interesting to determine whether injection of apoptotic thymocytes depleted for E1 ubiquitin enzymes to syngenic mice could induce autoimmunity, compared to injection of non-treated apoptotic thymocytes that should not induce autoimmunity (197). Another option would be to treat apoptotic extracts with non-specific DUB *in vitro*.

In conclusion, although it seems counterintuitive that modification of proteins by ubiquitin or UBLs may impinge on their visibility by the immune system, this unrecognized role of ubiquitin and UBL proteins in protecting from antigen-mediated detection by the immune system and its implication in immune tolerance could be a promising issue for the immunological field. Furthermore, this should highlight the therapeutic potential of manipulating E3 ligases and deubiquitinases in autoimmune diseases.

## Conflict of Interest Statement

The author declares that the research was conducted in the absence of any commercial or financial relationships that could be construed as a potential conflict of interest.
